# Violet LED light-activated MdHY5 positively regulates phenolic accumulation to inhibit fresh-cut apple fruit browning

**DOI:** 10.1093/hr/uhae276

**Published:** 2024-09-28

**Authors:** Juntong Jin, Liyong Qi, Shurong Shen, Shuran Yang, Hui Yuan, Aide Wang

**Affiliations:** Key Laboratory of Fruit Postharvest Biology (Liaoning Province), Key Laboratory of Protected Horticulture (Ministry of Education), National & Local Joint Engineering Research Center of Northern Horticultural Facilities Design & Application Technology (Liaoning), College of Horticulture, Shenyang Agricultural University, Shenyang 110866, China; Key Laboratory of Fruit Postharvest Biology (Liaoning Province), Key Laboratory of Protected Horticulture (Ministry of Education), National & Local Joint Engineering Research Center of Northern Horticultural Facilities Design & Application Technology (Liaoning), College of Horticulture, Shenyang Agricultural University, Shenyang 110866, China; Liaoning Agricultural Vocational and Technical College, Xiongyue 115009, China; Key Laboratory of Fruit Postharvest Biology (Liaoning Province), Key Laboratory of Protected Horticulture (Ministry of Education), National & Local Joint Engineering Research Center of Northern Horticultural Facilities Design & Application Technology (Liaoning), College of Horticulture, Shenyang Agricultural University, Shenyang 110866, China; Key Laboratory of Fruit Postharvest Biology (Liaoning Province), Key Laboratory of Protected Horticulture (Ministry of Education), National & Local Joint Engineering Research Center of Northern Horticultural Facilities Design & Application Technology (Liaoning), College of Horticulture, Shenyang Agricultural University, Shenyang 110866, China; Key Laboratory of Fruit Postharvest Biology (Liaoning Province), Key Laboratory of Protected Horticulture (Ministry of Education), National & Local Joint Engineering Research Center of Northern Horticultural Facilities Design & Application Technology (Liaoning), College of Horticulture, Shenyang Agricultural University, Shenyang 110866, China

## Abstract

Fresh-cut fruit browning severely affects the appearance of fruit. Light treatment can effectively inhibit fresh-cut apple fruit browning, but the regulatory mechanism remains unknown. Here, we discovered that violet LED (Light-Emitting-Diode) light treatment significantly reduced fresh-cut apple fruit browning. Metabolomic analysis revealed that violet LED light treatment enhanced the phenolic accumulation of fresh-cut apple fruit. Transcriptomic analysis showed that the expression of phenolic degradation genes *POLYPHENOL OXIDASE* (*MdPPO*) and *PEROXIDASE* (*MdPOD*) was reduced, and the expression of phenolic synthesis gene *PHENYLALANINE AMMONIA LYASE* (*MdPAL*) was activated by violet LED light treatment. Moreover, two *ELONGATED HYPOCOTYL 5* (*MdHY5* and *MdHYH*) transcription factors involved in light signaling were identified. The expression of *MdHY5* and *MdHYH* was activated by violet LED light treatment. Violet LED light treatment no longer inhibited fresh-cut apple fruit browning in MdHY5- or MdHYH- silenced fruit. Further experiments revealed that MdHY5 and MdHYH suppressed *MdPPO* and *MdPOD* expression and promoted *MdPAL* expression by binding to their promoters. In addition, MdHY5 and MdHYH bound to each other’s promoters and enhanced their expression. Overall, our findings revealed that violet LED light-activated *MdHY5* and *MdHYH* formed a positive transcriptional loop to regulate the transcription of *MdPPO*, *MdPOD*, and *MdPAL*, which in turn inhibited the degradation of phenolics and promoted the synthesis of phenolics, thus inhibiting fresh-cut apple fruit browning. These results provide a theoretical basis for improving the appearance and quality of fresh-cut apple fruit.

## Introduction

Fresh-cut fruit is popular in the market. It meets customers’ demands for freshness, nutrition, and convenience. Fresh-cut fruit accounts for 29% of total fruit consumption in Europe and the USA [1] and 11% in Japan and South Korea [2]. Although fresh-cut fruit occupies an essential position in the fruit market, many physiological and biochemical problems, especially browning, occur during the production of fresh-cut fruit, seriously reducing the quality and economic value of the fruit [3–5]. Fresh-cut fruit browning has been reported in various species, especially for white-fleshed fruit, including apple [6], pear [7], pitaya [8], melon [9], and pineapple [10]. Therefore, inhibiting the occurrence of browning is essential for making high-quality fresh-cut fruit.

Enzymatic browning is the main cause of fresh-cut fruit browning. In this process, phenolics are oxidized to produce quinones and interact with amino acids to form melanin [11]. As the substrate for enzymatic browning, phenolics are involved in the fresh-cut fruit browning reaction. On the other hand, phenolics, as the antioxidant substance, can scavenge free radicals and improve the antioxidant capacity of plants, maintaining the commercial value of fresh-cut products [12]. A recent report has shown that cold plasma inhibits fresh-cut mango browning by increasing total phenolics content [13]. Heat treatment enhances phenolic synthase PAL activity and total phenolics content to inhibit the browning of fresh-cut *Agaricus bisporus* [14]. Oxidative enzymes (PPO and POD) have been reported to play an important role in fresh-cut fruit browning [15, 16]. They can catalyze an array of phenolic compounds, resulting in browning. Silencing MdPPO in apple has been found to prevent discoloration after cutting [17]. Small DNA inserts have been found to reduce the expression of *PPO* and inhibit browning in potato [18]. H_2_S and plasma-processed air treatments prevent fresh-cut apple and lotus browning by reducing the activities of PPO and POD [19, 20].

As an important environmental factor, light regulates a series of fruit development processes. Light treatment is widely used in postharvest fresh-cut fruit storage due to its simple, low-cost, and pollution-free properties [21, 22]. A recent study confirms that pulsed light can effectively maintain fresh-cut apple’s quality and antioxidant characteristics and inhibit browning [23]. White fluorescent lamps can maintain the photosynthetic capacity of fresh-cut lettuce and reduce browning [24]. A study on button mushroom demonstrates that ultraviolet-C reduces PPO activity and *AbPPO* expression to inhibit browning [25]. Although light treatments are widely used to inhibit browning, the molecule mechanism by which light inhibits browning is not yet understood.

Transcription factors are involved in regulating many biological processes in plants. In light signaling, *elongated hypocotyl 5* (*HY5*), belonging to the basic leucine zipper domain (bZIP) transcription factor, acts downstream of various photoreceptors and mediates gene expression by directly interacting with light-responsive promoters [26]. Previous studies have confirmed that HY5 regulates plant growth, development, and environmental responses, including drought, salt stress, fruit ripening, and secondary metabolism [27]. For example, HY5 targets *B-BOX DOMAIN PROTEIN 7/8* (*BBX7/8*) to positively regulate cold acclimation after blue light treatment in *Arabidopsis thaliana* [28]. HY5 increases the accumulation of anthocyanins in tomato after blue light treatment by binding to the promoters of the anthocyanin synthesis genes [29]. A recent study demonstrates that HY5 binds to the apple’s *laccase 7 (MdLAC7)* promoter to regulate peel browning [30]. However, the role of HY5 in the browning of fresh-cut fruit has not been reported.

**Figure 1:**
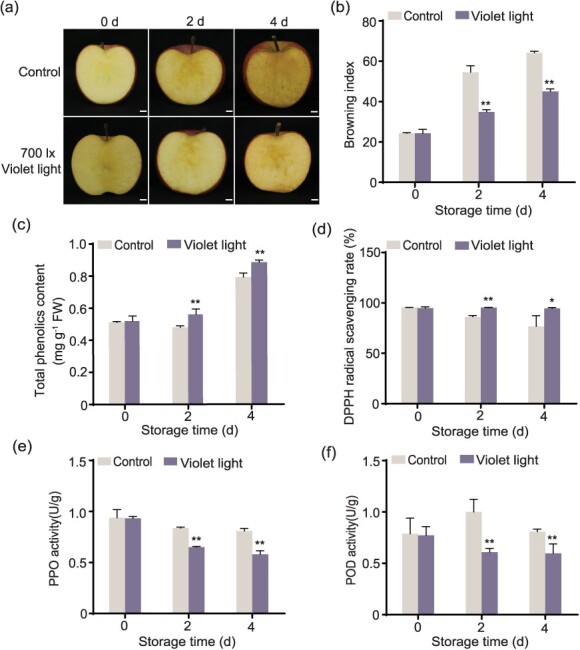
Violet LED light increased the antioxidant capacity and decreased oxidative enzyme activities of fresh-cut apple fruit. (a) ‘Fuji’ apple fruit were harvested in 2020 at 180 DAFB. Apple slices were treated with violet LED light at 700 lx at 10°C for 4 days, and samples were collected every 2 days. The samples were stored in the dark at 10°C as a control. Bars: 1 cm. BI (b), total phenolics content (c), DPPH radical scavenging rate (d), PPO activity (e), and POD activity (f) were investigated in the control and violet LED light-treated samples. The samples placed in the dark were used as a control. Data represents the means ± SE. Asterisks indicate significant differences (^*^P < 0.05; ^**^P < 0.01, Student’s *t-*test).

In this study, metabolome and transcriptome analyses were conducted to identify the differential metabolites and crucial genes involved in violet LED light inhibiting fresh-cut apple browning. Further investigation demonstrated that MdHY5 and MdHYH, which formed a positive transcriptional loop, regulated *MdPPO*, *MdPOD*, and *MdPAL* expression, thereby increasing phenolic accumulation and finally leading to a decrease in fresh-cut apple fruit browning. Overall, this study reveals the mechanism by which *MdHY5* and *MdHYH* mediate violet LED light-suppressed fresh-cut apple fruit browning. These results provide a new insight into the molecular basis of violet LED light inhibiting fresh-cut fruit browning.

## Results

### Violet LED light inhibited fresh-cut apple fruit browning

To clarify the role of light in fresh-cut apple fruit browning, we treated ‘Fuji’ apple slices with different light qualities, including red, orange, yellow, green, cyan, blue, violet, and white LED lights (Fig. S1), at 700 lx for 4 days. The result showed that orange, yellow, blue, violet, and white LED lights significantly inhibited fruit browning at both 2 and 4 days. The cyan LED light reduced browning only at 2 days. The red and green LED lights inhibited browning only at 4 days (Fig. 1a; Fig. S2a). Moreover, we found that only violet LED light significantly reduced the browning of ‘Hanfu’ apple fruit at both 2 and 4 days among all the lights (Fig. S2c). In the ‘Lvshuai’ apple, only yellow and violet LED lights significantly reduced fruit browning at both 2 and 4 days (Fig. S2e). The browning index (BI) showed the same result (Fig. 1b; Fig. S2b, d, f). These results indicated that the inhibition effect of violet LED light on the browning of fresh-cut apple fruit was more general than that of other lights.

To investigate the optimal light intensity, we applied 700, 1000, and 1500 lx violet LED lights to treat apple slices. We found that they all inhibited fresh-cut apple fruit browning (Fig. S3a, c, e), and the BI was significantly reduced by 700, 1000, and 1500 lx violet LED light treatments (Fig. S3b, d, f). Considering energy usage, 700 lx violet LED light was used for the following study.

A recent report has shown that antioxidant capacity negatively correlates with browning [30]. Here, we investigated apple’s antioxidant substances and activities after violet LED light treatment. We discovered that violet LED light treatment showed a higher total phenolics content than the control (Fig. 1c). DPPH radical scavenging rate was used to evaluate the antioxidant activity. We found that violet LED light treatment significantly increased the DPPH scavenging ability (Fig. 1d) and decreased the activities of oxidative enzymes PPO and POD (Fig. 1e, f). These results suggest that violet LED light might inhibit fresh-cut apple fruit browning by reducing oxidative enzyme activities and increasing antioxidant capacity.

### Differentially accumulated metabolites analysis under violet LED light

**Figure 2:**
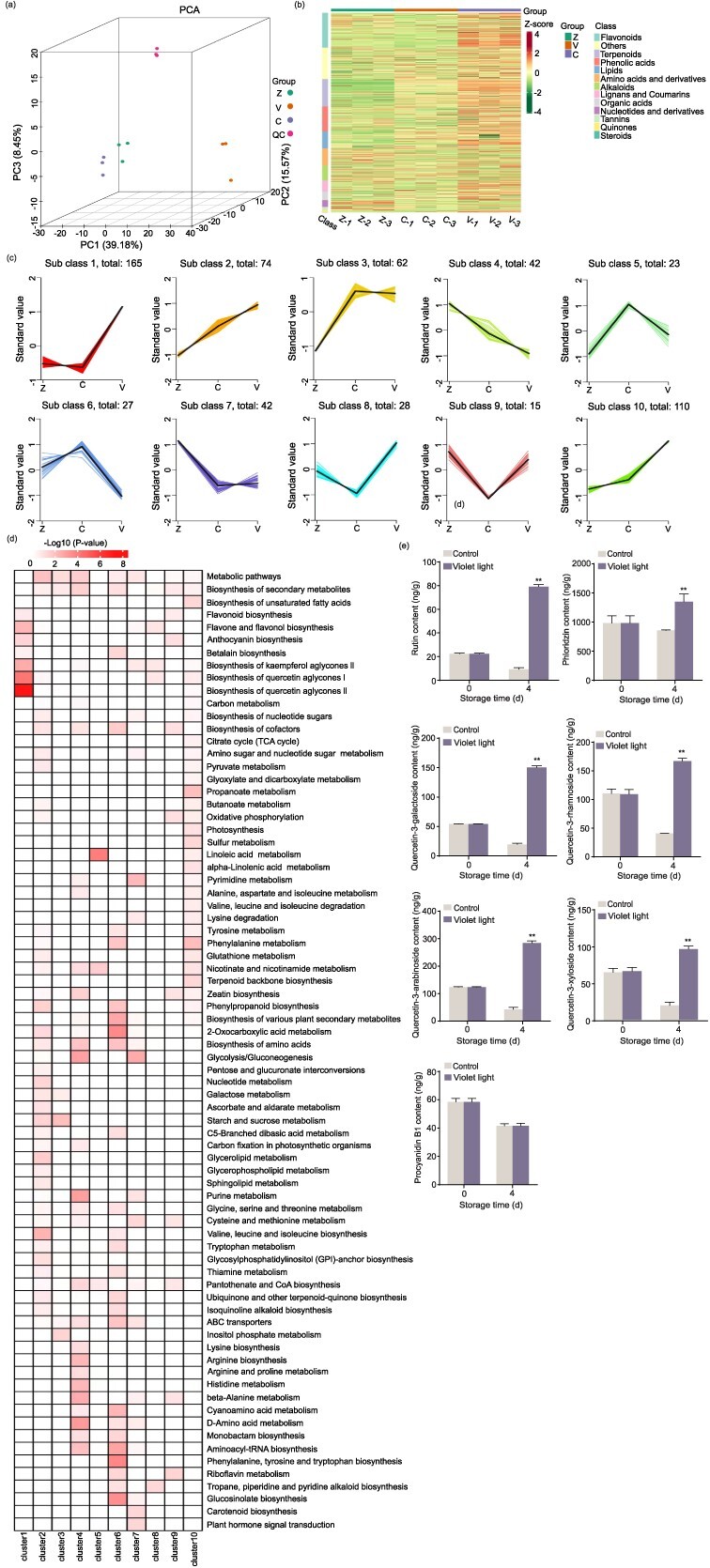
Overview of DAMs analysis. (a–d) The apple samples treated with or without violet LED light for 0 and 4 days were performed on metabolome analysis. Z represents the samples at 0 days. C represents the control samples at 4 days. V represents the violet LED light-treated samples at 4 days. Three replicates were conducted. (a) PCA analysis of metabolome. QC represents quality control. (b) Heat map analysis of differential compounds. (c) k-Means clustering analysis. The y-axis showed the Z-score of relative metabolite content. The numbers represented the numbers of DAMs in the clusters. (d) KEGG enrichment analysis of 10 clusters for DAMs. (e) The contents of rutin, phloridzin, quercetin-3-galactoside, quercetin-3-rhamnoside, quercetin-3-arabinoside, quercetin-3-xyloside, and procyanidin B1 were measured in the control and violet LED light-treated samples at 4 days. The samples placed in the dark were used as a control. Data represents the means ± SE. Asterisks indicate significant differences (^*^P < 0.05; ^**^P < 0.01, Student’s *t-*test).

To verify the changes of violet LED light on the metabolites of fresh-cut apple fruit, a widely targeted metabolome was conducted at two stages (0 and 4 days) with violet LED light-treated and control samples. Principal component analysis (PCA) revealed that metabolome data was highly reliable (Fig. 2a). In total, 390 differentially accumulated metabolites (DAMs) were identified in violet LED light-treated samples compared to control samples at 4 days (327 upregulated, 63 downregulated) (Fig. S4a). A total of 493 DAMs were identified in violet LED light-treated samples at 4 days compared to samples at 0 days (403 upregulated, 90 downregulated) (Fig. S4b). And 298 DAMs were identified in control samples at 4 days compared to samples at 0 days (196 upregulated, 102 downregulated) (Fig. S4c). Hierarchical cluster analysis showed that metabolites, especially phenolic acids and flavonoids, were upregulated in the samples treated with violet LED light for 4 days (Fig. 2b). K-means clustering analysis divided DAMs into 10 clusters (Fig. 2c). We observed that clusters 1 and 8 were correlated with the trend of browning. The relative metabolite content of clusters 1 and 8 was decreased in control samples at 4 days compared to samples at 0 days, and violet LED light treatment increased the relative metabolite content compared to control samples at 4 days. Moreover, an increased relative metabolite content was obtained in violet LED light-treated samples at 4 days compared to samples at 0 days. When researching the functions of these clustered metabolites, we found clusters 1 and 8 metabolites mainly enriched in phenylpropanoid and flavonoid biosynthesis pathways (Fig. 2d). These observed results demonstrate that violet LED light inhibits fresh-cut apple browning by inducing phenolic accumulation. Furthermore, to validate the metabolome data, several critical phenolic compounds in apple, including rutin, phloridzin, quercetin-3-galactoside, quercetin-3-rhamnoside, quercetin-3-arabinoside, quercetin-3-xyloside, and procyanidin B1 were detected by liquid chromatography–mass spectrometry (LC–MS) (Fig. 2e). The contents of these compounds (except procyanidin B1) were enhanced by violet LED light, which was consistent with the trend observed in metabolome. This result indicated the reliability of the metabolic data.

### Differentially expressed genes analysis under violet LED light

**Figure 3:**
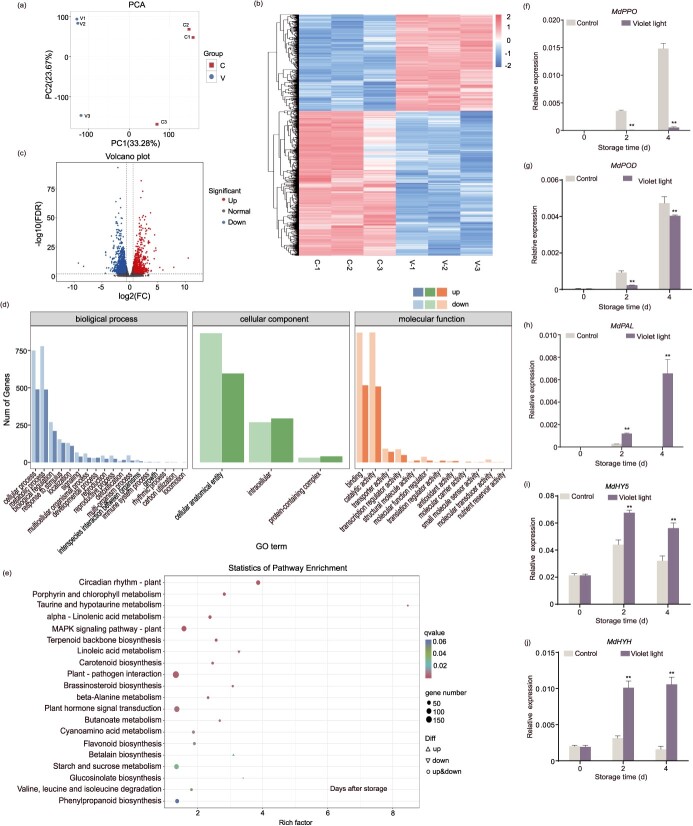
Overview of DEGs. (a–e) The apple samples treated with or without violet LED light for 4 days were performed on transcriptome analysis. Three replicates were conducted. C represents the control samples at 4 days. V represents the violet LED light-treated samples at 4 days. (a) PCA analysis of the transcriptome. (b) Hierarchical cluster analysis of DEGs. (c) Volcano plot analysis of DEGs. (d) GO analysis of DEGs. (e) KEGG enrichment pathway of DEGs. The expression levels of *MdPPO* (f), *MdPOD* (g), *MdPAL* (h), *MdHY5* (i), and *MdHYH* (j) were measured by qRT-PCR in the control and violet LED light-treated samples. The samples placed in the dark were used as a control. Data represents the means ± SE. Asterisks indicate significant differences (^*^P < 0.05; ^**^P < 0.01, Student’s *t-*test).

To investigate the mechanism of violet LED light inhibiting fresh-cut fruit browning, the transcriptome analysis was carried out with violet LED light-treated samples and control samples at 4 days. After quality control (Fig. 3a, b), 1397 upregulated genes and 2099 downregulated genes were identified (Fig. 3c). Gene ontology (GO) functional analysis showed these differentially expressed genes (DEGs) were enriched in molecular function, biological process, and cellular component (Fig. 3d). The KEGG pathways analysis indicated DEGs mainly enriched in phenylpropanoid and flavonoid biosynthesis pathways (Fig. 3e). It was in agreement with the enrichment of those observed DAMs in metabolome (Fig. 2d). Our investigation mainly focused on the potential vital genes involved in browning. We detected three genes, including two oxidative enzyme genes named *MdPPO* and *MdPOD*, and a phenolic synthase gene named *MdPAL* (Table. S1). qRT-PCR (Quantitative Real-time PCR) verified that *MdPPO* and *MdPOD* expression were downregulated, and *MdPAL* expression was upregulated by violet LED light treatment (Fig. 3f-h). Moreover, linear regression analysis showed that the expression levels of these genes were highly correlated with the BI, indicating that these genes might be critical in violet LED light inhibition of browning (Fig. S5).

Transcription factors are involved in regulating biological processes in plants [30]. Here, to clarify the role of transcription factors in violet light inhibition of fresh-cut fruit browning, we analyzed the transcriptome. A total of 95 differentially expressed transcription factors were identified, including *MYB*, *bHLH*, *WRKY*, *bZIP*, and *ERF*. Among them, we focused on transcription factors involved in the light signaling pathway and discovered two *HY5* transcription factors. We cloned these two HY5s and found one HY5 amino acid sequence was most similar to the AtHY5 protein, with 65% identity (Fig. S6), and another one was most similar to the AtHYH protein, with 42.13% identity (Fig. S7), so they were named *MdHY5* and *MdHYH*, respectively. *MdHY5* and *MdHYH* showed a higher expression level in violet LED light treatment than the control (Table. S2). qRT-PCR showed the same result (Fig. 3i, j). Chromosomal locations showed *MdHY5* and *MdHYH* sequences were located on chromosomes 15A and 16A, respectively, according to the haplotype-resolved genome of ‘Fuji’ (Table. S3). Amino acid sequence analysis with DNAMAN software showed that MdHY5 and MdHYH only had 35.53% identity (Fig. S8). Furthermore, a phylogenetic analysis between MdHY5, MdHYH, and their homologs in apple and other species was carried out. HY5s protein sequences were obtained according to Burman [31] and Wang [32]. The result indicated that MdHY5 and MdHYH belonged to different clades (Fig. S9). Correlation analysis showed that *MdHY5* and *MdHYH* expression levels were highly correlated with the expression of *MdPPO*, *MdPOD*, *MdPAL*, and the BI (Fig. S10).

### *MdHY5* and *MdHYH* were essential for violet LED light inhibiting fresh-cut apple fruit browning

**Figure 4:**
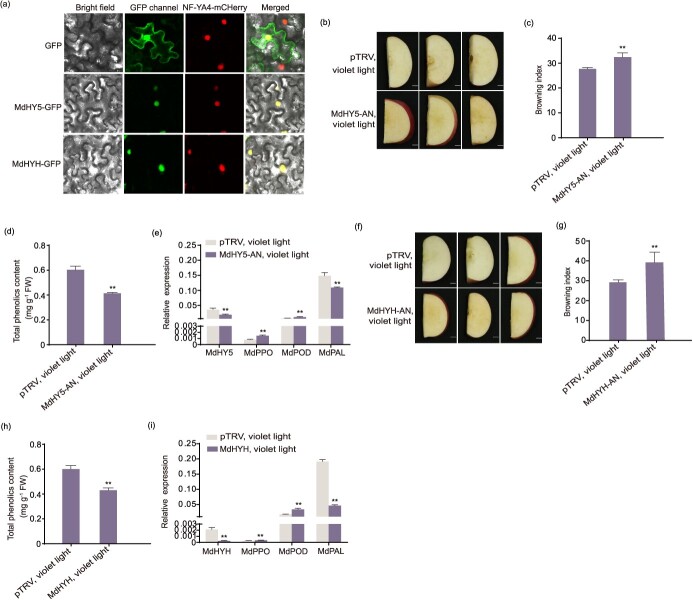
*MdHY5* and *MdHYH* were required for violet LED light inhibiting fresh-cut apple fruit browning. (a) Subcellular localization of MdHY5 and MdHYH. *MdHY5* and *MdHYH* CDS were cloned in pRI101 vectors containing GFP controlled by *35S* promoter. Then, vectors were introduced into *A. tumefaciens* strain EHA105 and injected with *N. benthamiana* leaves transiently. The injected leaves were observed after 2 days. NF-YA4-mCherry was used as a nuclear marker. Leaves infiltrated with empty GFP vectors were used as a control. Scale bars, 25 μm. (b–e) MdHY5 was silenced in apple slices by transient infection. MdHY5-AN fruit was then treated with violet LED light for 4 days. Fruit infiltrated with empty vector (pTRV) was placed under violet LED light for 4 days as a control. Scale bars, 1 cm. The BI (c) and the total phenolics content (d) were measured. (e) The expression levels of *MdHY5*, *MdPPO*, *MdPOD*, and *MdPAL* were investigated by qRT-PCR in MdHY5-AN and control fruit after violet LED light treatment. (f–i) Transient infection was used to silence MdHYH in apple slices, and MdHYH-AN fruit was treated with violet LED light for 4 days. Fruit infiltrated with empty vector (pTRV) was also placed under violet LED light for 4 days as a control. Scale bars, 1 cm. The BI (g) and the total phenolics content (h) were measured. (i) The expression levels of *MdHYH*, *MdPPO*, *MdPOD*, and *MdPAL* were evaluated in MdHYH-AN and control fruit after violet LED light treatment. The experiment was performed independently in three biological replicates. Data represents the means ± SE. Asterisks indicate significant differences (^*^P < 0.05; ^**^P < 0.01, Student’s *t-*test).

To investigate the functions of *MdHY5* and *MdHYH* in violet LED light inhibiting fresh-cut apple fruit browning, MdHY5-GFP and MdHYH-GFP fusion proteins were separately overexpressed in *Nicotiana benthamiana* leaves. Microscopy showed that the MdHY5-GFP and MdHYH-GFP proteins both localized to the nucleus (Fig. 4a). Next, to investigate M*dHY5* and *MdHYH* functions in apple, we transiently silenced *MdHY5* and *MdHYH* in apple, respectively. Then, violet LED light treated fruit for 4 days at 10°C. The results showed that violet LED light treatment no longer inhibited the browning of fresh-cut apple fruit in MdHY5-AN or MdHYH-AN fruit (Fig. 4b, f). A significantly enhanced BI was obtained in MdHY5-AN or MdHYH-AN fruit after violet LED light treatment (Fig. 4c, g). The total phenolics content was decreased in MdHY5-AN or MdHYH-AN fruit after violet LED light treatment (Fig. 4d, h). qRT-PCR demonstrated that the expression levels of *MdPPO* and *MdPOD* were higher, and the expression level of *MdPAL* was lower in MdHY5-AN or MdHYH-AN fruit than the control after violet LED light treatment (Fig. 4e, i), suggesting MdHY5 and MdHYH might regulate *MdPPO*, *MdPOD*, *MdPAL* expression to inhibit browning. These results demonstrate the importance of *MdHY5* and *MdHYH* in violet LED light inhibiting fresh-cut apple fruit browning.

**Figure 5:**
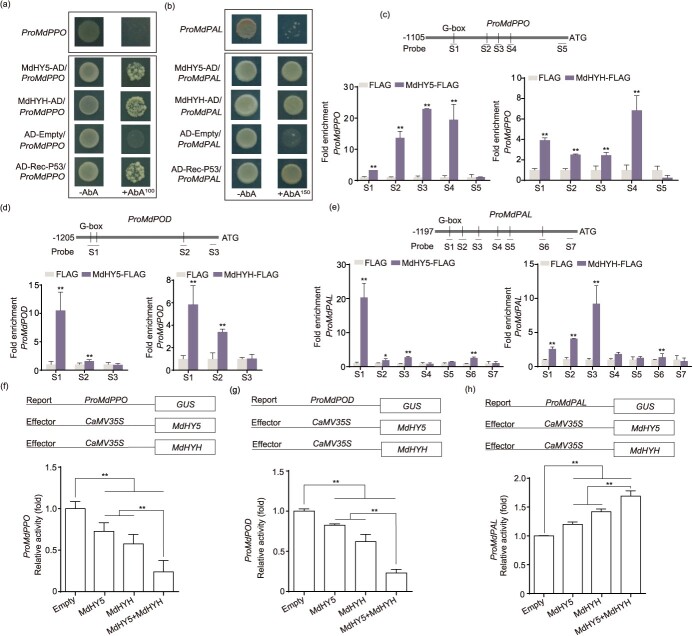
MdHY5 and MdHYH regulated *MdPPO*, *MdPOD*, and *MdPAL* expression. (a, b) Y1H assay showed that MdHY5 and MdHYH bound to the *MdPPO* and *MdPAL* promoters. P53 acted as a positive control. Empty vector pGADT7 (AD) worked as a negative control. (c–e) ChIP-PCR analysis showed that MdHY5 and MdHYH bound to the *MdPPO*, *MdPOD*, and *MdPAL* promoters *in vivo*. The FLAG antibody was used to verify the binding of MdHY5 and MdHYH to the *MdPPO*, *MdPOD*, and *MdPAL* promoters, and the results were detected through qPCR. Fruit calli with a FLAG tag alone was used as a negative control. The experiment was performed independently in three biological replicates. (f–h) GUS activation assay showed that MdHY5 and MdHYH negatively regulated the *MdPPO* and *MdPOD* promoters and positively regulated the *MdPAL* promoter. The experiment was performed independently in three biological replicates. Data represents the means ± SE. Asterisks indicate significant differences (^*^P < 0.05; ^**^P < 0.01, Student’s *t-*test).

### MdHY5 and MdHYH bound to *MdPPO*, *MdPOD*, and *MdPAL* promoters and regulated their transcription

To verify whether MdHY5 and MdHYH could regulate the transcription of *MdPPO*, *MdPOD*, and *MdPAL*, we analyzed their promoters and found several G-boxes (HY5 binding site) in the *MdPPO*, *MdPOD*, and *MdPAL* promoters. Yeast one-hybrid (Y1H) assay showed that MdHY5 and MdHYH directly bound to the *MdPPO* and *MdPAL* promoters (Fig. 5a, b). Y1H assay was not performed on the *MdPOD* promoter because its promoter could not be inhibited by AbA^1000^ on the SD/-Ura medium (Fig. S11). To verify whether MdHY5 and MdHYH could bind to the G-box in the *MdPPO*, *MdPOD*, and *MdPAL* promoters, we performed an electrophoretic mobility shift assay (EMSA). The first fragment in the promoter containing the G-box binding site was selected to generate a hot probe. The result indicated that MdHY5 and MdHYH bound to the G-box in the *MdPPO*, *MdPOD*, and *MdPAL* promoters, and the addition of unlabeled competing probes weakened the binding bands. Treatment with thermal mutation probes inhibited the binding of MdHY5 and MdHYH to the promoters (Fig. S12). These results demonstrate that MdHY5 and MdHYH bind to the *MdPPO*, *MdPOD*, and *MdPAL* promoters *in vitro*. Next, we performed a Chromatin immunoprecipitation (ChIP)-qPCR assay for *in vivo* validation. The result showed that *MdPPO*, *MdPOD*, and *MdPAL* promoters were enriched in the *MdHY5* and *MdHYH* overexpressing apple calli (Fig. 5c–e), indicating that MdHY5 and MdHYH also bound to *MdPPO*, *MdPOD*, and *MdPAL* promoters *in vivo*.

**Figure 6:**
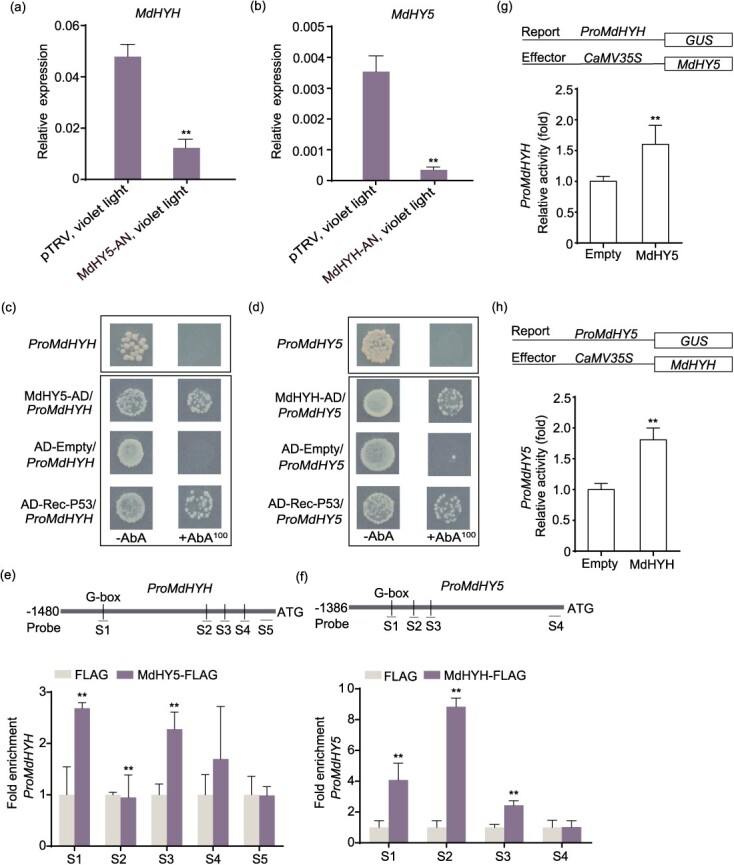
MdHY5 and MdHYH positively regulated each other’s promoters. (a, b) The expression levels of *MdHYH* and *MdHY5* in MdHY5-AN and MdHYH-AN fruit after violet LED light treatment. (c, d) Y1H analysis showed that MdHY5 and MdHYH bound to each other’s promoters. P53 acted as a positive control. Empty vector pGADT7 (AD) worked as a negative control. (e, f) ChIP-PCR analysis showed that MdHY5 and MdHYH bound to each other’s promoters *in vivo*. The FLAG antibody was used to verify the binding of MdHY5 and MdHYH to each other’s promoters, and the result was detected through qPCR. Fruit calli with a FLAG tag alone was used as a negative control. The experiment was performed independently in three biological replicates. (g, h) The GUS activation assay showed that MdHY5 positively regulated the *MdHYH* promoter and MdHYH positively regulated the *MdHY5* promoter. The experiment was performed independently in three biological replicates. Data represents the means ± SE. Asterisks indicate significant differences (^*^P < 0.05; ^**^P < 0.01, Student’s *t-*test).

We then investigated the regulation of *MdPPO*, *MdPOD*, and *MdPAL* by MdHY5 and MdHYH using a β-glucuronidase (GUS) activity assay in *N*. *benthamiana* leaves. When *Pro35S: MdHY5/MdHYH* was co-expressed with *ProMdPPO:GUS* or *ProMdPOD:GUS*, a significantly reduced GUS activity was observed compared with the control, indicating that MdHY5 and MdHYH suppress the activities of *MdPPO* and *MdPOD* promoters (Fig. 5f, g). Co-expression of *Pro35S:MdHY5/MdHYH* and *ProMdPAL:GUS* significantly enhanced GUS activity compared with the control, indicating that MdHY5 and MdHYH activate the activity of *MdPAL* promoter (Fig. 5h). We then found that the co-existence of MdHY5 and MdHYH significantly inhibited the activities of *MdPPO* and *MdPOD* promoters and increased the activity of the *MdPAL* promoter compared with either of the two alone (Fig. 5f–h). Thus, we speculate that MdHY5 and MdHYH play an independent role in inhibiting the browning process.

### MdHY5 and MdHYH promoted each other’s expression via interaction with corresponding promoters

In MdHY5-silenced fruit, we found the expression of *MdHYH* was significantly decreased by violet LED light treatment. Similarly, we found that *MdHY5* expression was reduced in MdHYH-silenced fruit by violet LED light treatment (Fig. 6a, b). Therefore, we speculate that there may be a regulatory relationship between *MdHY5* and *MdHYH*. Y1H and EMSA assays indicated that MdHY5 and MdHYH bound to each other’s promoters (Fig. 6c, d and Fig. S13). ChIP-qPCR showed that MdHYH and MdHY5 promoters were separately enriched in the *MdHY5* and *MdHYH* overexpressing apple calli (Fig. 6e, f). These results indicate that MdHY5 and MdHYH bind to each other’s promoters both *in vitro* and *in vivo*.

We then co-expressed *Pro35S:MdHY5* with *ProMdHYH:GUS* in *N*. *benthamiana* leaves and found that GUS activity significantly increased compared with the control. The same result was obtained when *Pro35S: MdHYH* was co-expressed with *ProMdHY5:GUS* in *N*. *benthamiana* leaves (Fig. 6g, h). These results indicate that MdHY5 and MdHYH activate each other’s promoters.

## Discussion

The browning of fresh-cut fruit during processing seriously reduces fruit quality and economic value. Light has been widely reported to inhibit fresh-cut fruit browning, but these studies only investigated the effect of light on the browning of fresh-cut fruit and the expression profile and activity of genes involved in browning [21]. The molecular mechanism by which light inhibits fresh-cut fruit browning is unclear.

The enzymatic browning of fruit is a complex physiological and biochemical process. When plant cells are destroyed, phenolics are oxidized to form quinones, which results in browning [11]. Phenolics serve not only as oxidation substrates for enzymes but also as antioxidant substances to resist free radical damage. Phenolics can directly scavenge H_2_O_2_ and reduce the accumulation of reactive oxygen species [33]. In plants, total phenolics content depends on the balance between biosynthesis and conversion. In other words, the observed increase in the phenolics content indicates that the synthesis rate is higher than the conversion rate. At this time, the antioxidant capacity of phenolics is playing a significant role. While a decrease in the phenolics content indicates that phenolics mostly have been converted into quinones [34]. In this study, increased phenolics content and decreased oxidative enzyme activities and expression after violet LED light treatment (Fig. 1 and 2) indicated that violet LED light treatment promoted phenolic synthesis rate and inhibited phenolic conversion rate.

The effect of light on plants depends on light quality and light intensity [35]. Studies have shown that blue light regulates different plant processes, such as stomatal opening, photosynthesis, and photo-morphogenesis [36, 37]. Red light affects seed germination as well as the growth and flowering of plants [38, 39]. For example, blue light promotes the photosynthetic capacity of leaves and is beneficial for cucumber growth [40]. In strawberry, blue light is superior to white and red light in inducing anthocyanin synthesis [41]. For berry, violet LED light is the most useful for increasing the content of total phenolics and vitamin C compared with yellow and blue light [42]. In this study, we observed that the violet LED light was better than others in inhibiting fresh-cut apple fruit browning (Fig. 1a, b and Fig. S2). We found that orange, blue, and white light also inhibited browning in ‘Fuji’ fruit at 2 and 4 days. However, in ‘Hanfu’ and ‘Lvshuai’ apple, the inhibitory effect was insignificant, indicating that they were not crucial to browning inhibition. We speculate that they may be mainly involved in other metabolism processes.

Plants perceive light signals from different photoreceptors and converge on the downstream transcription factor HY5 [43, 44]. HY5 integrates light signals with other processes, such as hormone signaling and nutrient accumulation [45, 46]. For example, SiHY5 promoted fruit ripening and increased the carotenoid content in tomato [47]. A more recent study showed that MdHY5 negatively regulated the expression of *MdWRKY31*, which further inhibited *MdLAC7* expression and peel browning in apple [30]. In this study, MdHY5 and MdHYH were shown to integrate violet LED light signal with fresh-cut apple fruit browning process, revealing a new function of HY5 (Fig. 4). Interestingly, we found that MdHY5 and MdHYH positively regulated *MdPAL* expression and negatively regulated *MdPPO* and *MdPOD* expression, suggesting that they could play different roles in the browning process (Fig. 5). Similarly, a recent report shows that *MYB306-like* plays various roles in regulating anthocyanin accumulation in apple [48]. However, the mechanism by which HY5 plays two distinct roles in the same process is unclear and needs further study.

Finally, this work showed that violet LED light-activated MdHY5 and MdHYH promoted each other’s expression, weakened *MdPPO* and *MdPOD* transcription, and enhanced *MdPAL* transcription, which resulted in an increase in phenolic content and a decrease in browning. In the dark, the expression levels of *MdHY5* and *MdHYH* were suppressed, and the ability of *MdHY5* and *MdHYH* to regulate downstream genes was reduced, resulting in browning.

## Materials and methods

### Plant material and treatments

‘Fuji’, ‘Hanfu’, and ‘Lvshuai’ apple fruit (*Malus* × *domestica* Borkh.) were obtained from an experimental farm at the Liaoning Pomology Institute (Xiongyue, China) in 2020. ‘Fuji’ and ‘Hanfu’ apple fruit were collected at 180 days after full bloom (DAFB); ‘Lvshuai’ apple fruit were collected at 120 DAFB. Apple fruit were transferred immediately to the laboratory and stored in a refrigerator for testing. Multicolored climate chambers were used to irradiate fresh-cut apple fruit. The spectra and wavelengths of different lights were measured by the spectrometer (Lighting Passport, Taiwan, China). Apple fruit were cut into slices and divided into nine groups (25 slices per group), of which eight groups were sequentially treated with 641 nm red, 600 nm orange, 592 nm yellow, 515 nm green, 468 nm cyan, 457 nm blue, 433 nm violet, and white LED light, with a 579-nm dominant wavelength. The ninth group was placed directly in the dark as a control. The light intensity was set to 700, 1000, and 1500 lx. All apple slices were placed at 10°C for 4 days. Ten slices were frozen in liquid nitrogen every 2 days and stored at −80°C for further use.

### Measurement of the BI

The flesh color of fresh-cut fruit was measured using a chroma meter (Chroma Meter CR-400, Tokyo, Japan), and L^*^, a^*^, and b^*^ values for the fruit surface were recorded [8]. The BI was calculated using the following equations: BI = (x − 0.31) × 100/0.172, where x = (a^*^ + 1.75 × L^*^)/(5.645 × L^*^ + a^*^ − 3.012 × b^*^).

### Measurement of PPO and POD activities

The PPO and POD activities were measured according to the method of Simões et al [49]. Apple fruit samples (0.5 g) were homogenized with 2 ml sodium phosphate buffer (0.2 M, pH = 6.0) and centrifuged at 10 000 g for 21 min at 4°C. The supernatant obtained after centrifugation was the enzymatic extract.

For the PPO enzyme activity assay, 500 μl enzymatic extract and 500 μl catechin (0.2 mol/l) were added to 500 μl phosphoric acid buffer (pH 6.5). The absorbance at 420 nm was measured every 30 s for 2 min.

For the POD enzyme activity assay, 100 μl enzymatic extract was added to the reaction mixture containing 500 μl sodium phosphate buffer (0.2 mol l^−1^, pH 6.0), 200 μl guaiacol (0.5%), and 200 μl H_2_O_2_ (0.08%). The absorbance at 470 nm was measured every 30 s for 2 min.

The result was expressed as the unit of U/g fresh weight. One unit of PPO or POD activity was defined as a change of 0.01 per min.

### Measurement of total phenolics content and phenolic components

Apple fruit samples (0.5 g) were extracted by homogenization with 5 ml 80% (v/v) methanol and centrifuged at 10 000 g for 20 min at 4°C. The total phenolics content was measured according to the method of Fan et al [50]. Phenolic components were determined using LC–MS. The method was described by Zhang et al [51].

### Measurement of total antioxidant activity

The total antioxidant activity of the samples was measured by the 1.1-diphenyl-2-picrylhydrazyl radicals (DPPH) method [52].

### Widely targeted metabolome analysis

Metabolome analysis of the apple sample treated with or without violet LED light for 0 and 4 days was performed by MetWare Biological Science and Technology Co., Ltd. (Wuhan, China). The DAMs were identified by fold change ≥1.5 and a variable influence on projection (VIP) ≥ 1. Metabolomics data was listed in the Supplemental table (Table S4–S6).

### Transcriptome sequencing

Transcriptome analysis of the apple samples treated with or without violet LED light for 4 days was performed by Biomarker Technologies (Beijing, China). The method of transcriptome sequencing was performed as previously described [53]. The sequencing libraries were sequenced on an Illumina NovaSeq platform. The raw data reads were obtained by the BMKCloud online platform (www.biocloud.net). The clean data was filtered from the raw data by the in-house script to remove adapters, primer sequences, low-quality reads, and reads containing poly (N). Clean reads were aligned to the ‘Fuji’ genome (https://figshare.com/articles/dataset/The_chromosome-level_haploid_genom-e_Assembly_of_Malus_domestica_Fuji_/23803938). The DEGs were identified by fold change ≥1.5. All the raw reads have been submitted to NCBI under accession number PRJNA1124363.

### RNA extraction and gene expression analysis

Total RNA was extracted using the method of Li et al [54]. Gene expression was performed using qRT-PCR [55]. Apple *ACTIN* (EB136338) was used as an internal reference gene.

### Phylogenetic tree construction

The MEGA 11 [56] program was used to construct the phylogenetic tree. Sequence data of MdHY5s can be found in the ‘Fuji’ genome. Sequence data of AtHY5s can be found in the TAIR (http://www.arabidopsis.org). Other sequence data can be found in the PLAZA (http://bioinformatics.psb.ugent.be/plaza/versions/plaza _v3_dicots).

### Subcellular localization of MdHY5 and MdHYH

*MdHY5* and *MdHYH* CDS were separately ligated into a pRI101 vector containing a GFP tag (*35S: GFP-MdHY5*, *35S: GFP-MdHYH*). MdHY5-GFP or MdHYH-GFP injection buffer was injected into *N*. *benthamiana* leaves, and leaves infiltrated with empty GFP vector were used as a control. A confocal microscope was used to observe the fluorescence after 3 days, and NF-YA4-mCherry was used as a nuclear marker [57].

### *Agrobacterium*-mediated infiltration

To overexpress *MdHY5* and *MdHYH* in apple calli (Orin), full-length *MdHY5* and *MdHYH* were ligated separately into upstream of 3x FLAG in the pRI101 vector with *35S* to form *Pro35S: MdHY5-FLAG* and *Pro35S: MdHYH-FLAG*. The *Agrobacterium tumefaciens* EHA105 strain containing *Pro35S: MdHY5-FLAG* or *MdHYH-FLAG* was resuspended in 50 ml MS liquid medium with 1 mM acetosyringone to generate the infection buffer. Apple calli was put in the infection buffer for 20 min, collected, spread on MS solid medium, and cultivated for 3 days.

To silence *MdHY5* and *MdHYH* in apple slices, respectively, partial *MdHY5* and *MdHYH* CDS (1–300 bp) were ligated into the pTRV2 vector to form MdHY5-AN and MdHYH-AN vectors. The sequences of MdHY5-AN and MdHYH-AN were listed in the Supplemental Table 7. The infiltration buffer was prepared as previously described [54]. Apple slice infiltration was performed as previously described [58], with slight modifications. Briefly, fresh-cut apple slices were incubated in the infiltration buffer for 1 h. Then, infiltration buffer was injected into apple slices under a −70 kPa vacuum. The injected samples were immediately placed in a light incubator and treated with violet LED light at 10°C for 4 days.

### Y1H assay

*MdHY5* and *MdHYH* CDS were separately cloned into the pGADT7 vector. *MdPPO*, *MdPOD*, and *MdPAL* promoter fragments were ligated separately into the pAbAi vector. The Y1H assay was performed as previously described [54].

### EMSA

*MdHY5* and *MdHYH* CDS were separately ligated into the pEASY-E1 vector (catalog no. CT101–01; TransGen Biotech, Beijing, China) and transformed into competent *Escherichia coli* BL21 (DE3) cells (TransGen Biotech). Proteins were then purified using the method described by Li et al [55]. For EMSA, a 3′-biotin-end-labeled double-stranded DNA probe was prepared. EMSA was performed using a Light Shift Chemiluminescence EMSA Kit (catalog no. GS009; Beyotime, Shanghai, China), as previously described [54].

### ChIP-PCR analysis

*MdHY5* and *MdHYH* CDS were separately cloned into the pRI101 vector with a 3x FLAG tag. The ChIP assay was performed using a ChIP kit (catalog no. 56383; Cell Signaling Technology, Danvers, MA, USA) according to the manufacturer’s instructions. The FLAG (catalog no. YM3808; ImmunoWay, California, America) antibody was used to verify the binding of MdHY5 and MdHYH to the *MdPPO*, *MdPOD*, and *MdPAL* promoters and the enrichment of the immunoprecipitate was analyzed using qPCR. The calli was infected three times, and three ChIP assays were performed as three biological replicates.

### GUS activation assay

The *MdPPO*, *MdPOD*, and *MdPAL* promoters were separately inserted into the upstream region of the *GUS* reporter gene in the pBI121 vector as a reporter. *MdHY5* and *MdHYH* CDS were separately cloned into the pRI101 overexpression vector as effectors. Finally, the reporter and effector were co-injected into *N*. *benthamiana* leaves. GUS activity was measured using the method described by Li et al [54].

## Supplementary Material

Web_Material_uhae276

## Data Availability

Transcriptome data during the study were deposited in the NCBI under accession number PRJNA1124363. Sequence data from this study can be found in the ‘Fuji’ genome (https://figshare.com/articles/dataset/The_chromosome-level_haploid_genom-e_Assembly_of_Malus_domestica_Fuji_/23803938) or the GenBank libraries under accession numbers: *MdPPO* (FujiC05BgG031160), *MdPOD* (FujiC14BgG000850), *MdPAL* (FujiC04BgG009830), *MdHY5* (FujiC15AgG000420), *MdHYH* (FujiC16AgG011950), and *MdActin* (EB136338).
