# Supplementary material for: Violet LED light-activated MdHY5 positively regulates phenolic accumulation to inhibit fresh-cut apple fruit browning
Source: Hortic Res. 2024 Sep 28;12(1):uhae276. doi: 10.1093/hr/uhae276 (PMC11739620; doi:10.1093/hr/uhae276)
Supplement: Web_Material_uhae276 [file web_material_uhae276.zip › Supplemental Figure.docx]

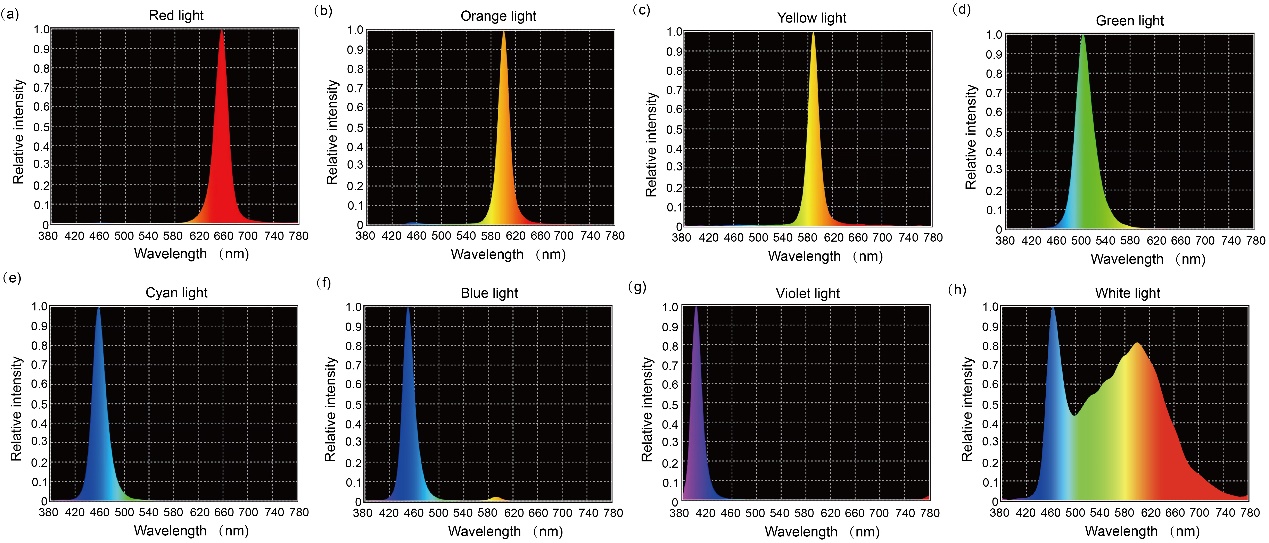


**Supplemental Figure S1.** Spectra and wavelengths of different lights.

(a) The wavelength of red light was 641 nm, (b) orange light was 600 nm, (c) yellow light was 592 nm, (d) green light was 515 nm, (e) cyan light was 468 nm, (f) blue light was 457 nm, (g) violet light was 433 nm, (h) white light was a mixture of all visible lights (400-720 nm), 579 nm was the dominant wavelength within the mixture. The Y-axis represents the relative intensity. Relative intensity indicates the radiant energy of different wavelengths of light in the spectrum.


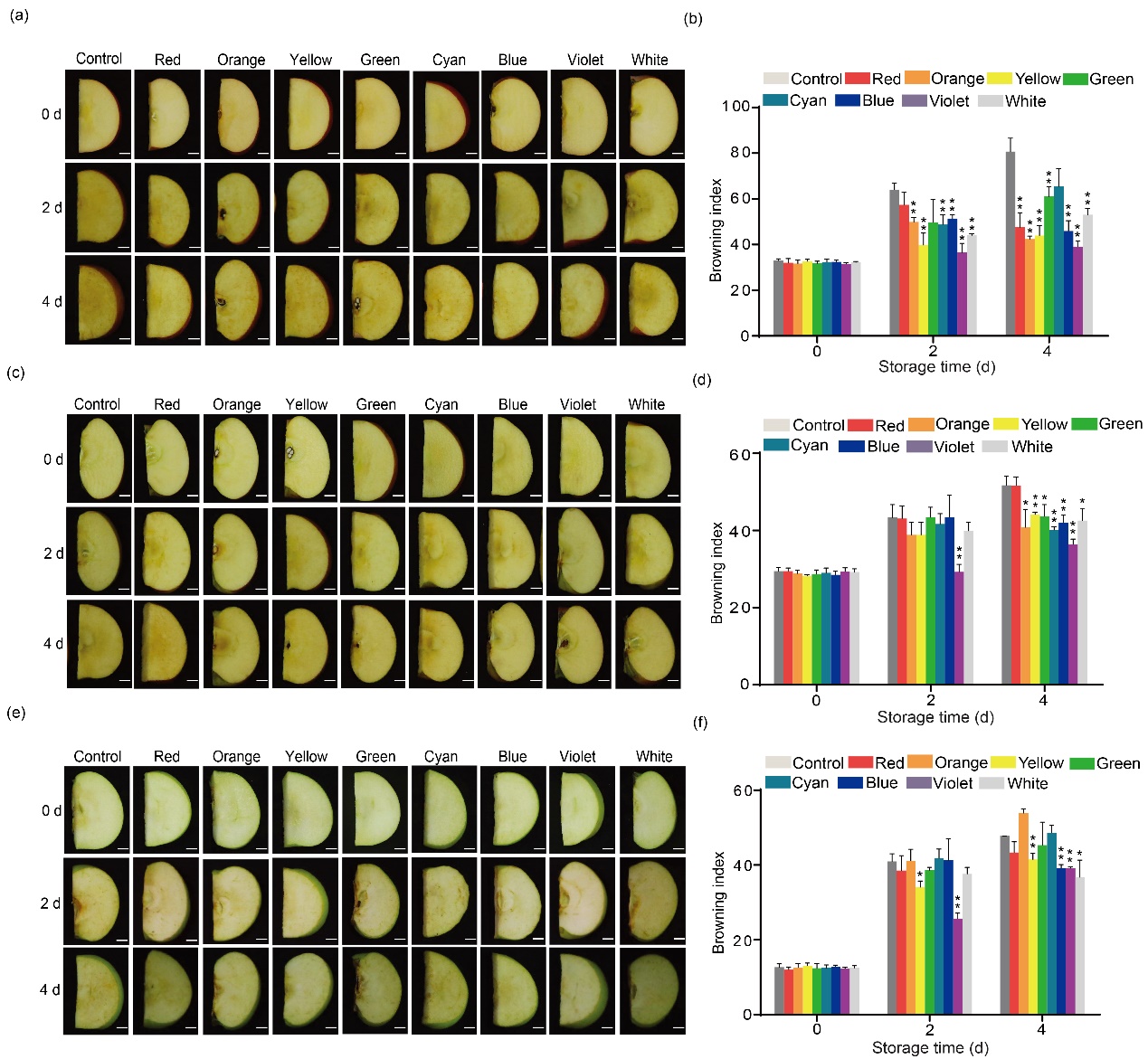


**Supplemental Figure S2.** Different light qualities inhibited fresh-cut apple fruit browning.

(a) ‘Fuji’ apple fruit were harvested at 180 days after full bloom (DAFB) in 2020 and stored at 4°C for later use. Samples were treated with red, orange, yellow, green, cyan, blue, violet and white LED lights with 700 lx at 10°C for 4 d. Samples were collected every 2 d. Dark was used as control. Bars, 1cm. (b) Browning index was measured at 2 d and 4 d in the control samples and 700 lx LED light-treated ‘Fuji’ samples by the chroma meter (Chroma Meter CR-400, Tokyo, Japan). (c) ‘Hanfu’ apple fruit were harvested at 180 DAFB in 2020 and stored at 4°C for later use. Samples were treated with red, orange, yellow, green, cyan, blue, violet and white LED lights with 700 lx at 10°C for 4 d. Samples were collected every 2 d. Dark was used as control. Bars, 1cm. (d) Browning index was measured at 2 d and 4 d in the control samples and 700 lx LED light-treated ‘Hanfu’ samples. (e) ‘Lvshuai’ apple fruit were harvested at 120 DAFB in 2020 and stored at 4°C for later use. Samples were treated with red, orange, yellow, green, cyan, blue, violet and white LED lights with 700 lx at 10°C for 4 d. Samples were collected every 2 d. Dark was used as control. Bars, 1cm. (f) Browning index was measured at 2 d and 4 d in the control samples and 700 lx LED light-treated ‘Lvshuai’ samples. Three biological replicates were analyzed. Values represent means ± SE. Asterisks indicate significant differences (*P < 0.05; **P < 0.01, Student’s *t-*test).


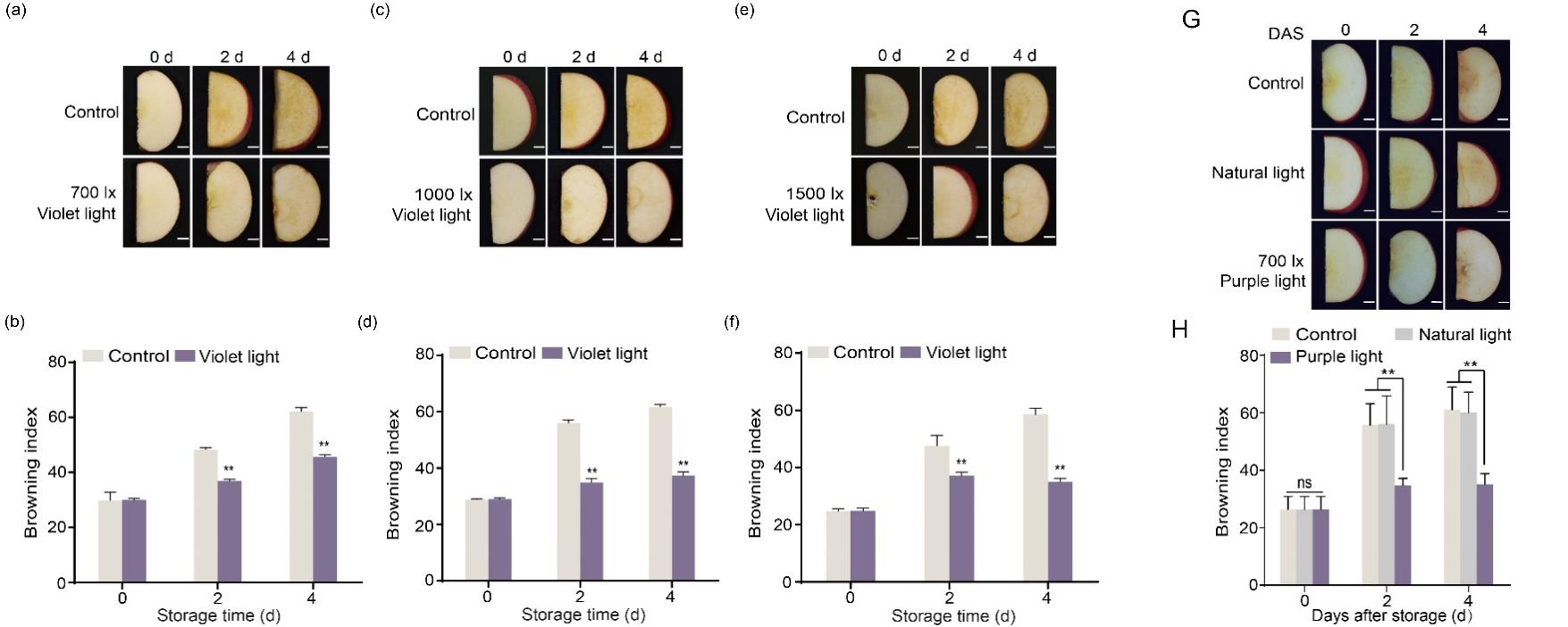


**Supplemental Figure S3.** Violet LED light of different light intensities inhibited fresh-cut ‘Fuji’ apple fruit browning.

(a) ‘Fuji’ apple fruit were treated with 700 lx violet LED light at 10°C for 4 d. Samples were collected every 2 d. Dark was used as a control. Bars, 1cm. (b) Browning index was measured in the control samples and 700 lx violet light-treated samples at 2 d and 4 d. (c) ‘Fuji’ apple fruit were treated with 1000 lx violet LED light at 10°C for 4 d. Samples were collected every 2 d. Dark was used as a control. Bars, 1cm. (d) Browning index was measured in the control samples and 1000 lx violet light-treated samples at 2 d and 4 d. (e) ‘Fuji’ apple fruit were treated with 1500 lx violet LED light at 10°C for 4 d. Samples were collected every 2 d. Dark was used as a control. Bars, 1cm. (f) Browning index was measured in the control samples and 1500 lx violet light-treated samples at 2 d and 4 d. Three biological replicates were analyzed. Values represent means ± SE. Asterisks indicate significant differences as determined by Student’s *t-*test (*P < 0.05; **P < 0.01, Student’s *t-*test).


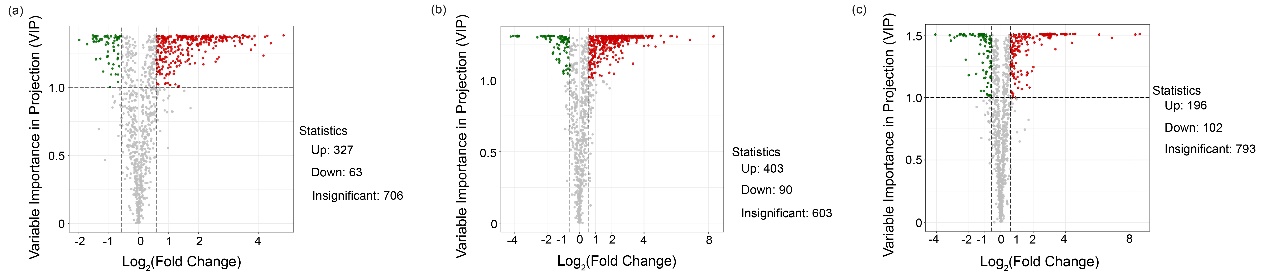


**Supplemental Figure S4.** Differential metabolites analysis.

(a) Volcano plot showed 327 differential metabolites were upregulated and 63 differential metabolites were downregulated in violet LED light-treated samples compared to control samples at 4 d. (b) Volcano plot showed 403 differential metabolites were upregulated and 90 differential metabolites were downregulated in violet LED light-treated samples at 4 d compared to samples at 0 d. (c) Volcano plot showed 196 differential metabolites were upregulated and 102 differential metabolites were downregulated in control samples at 4 d compared to samples at 0 d. Green points represent downregulated metabolites. Red points represent upregulated metabolites. Gray points represent insignificant metabolites.


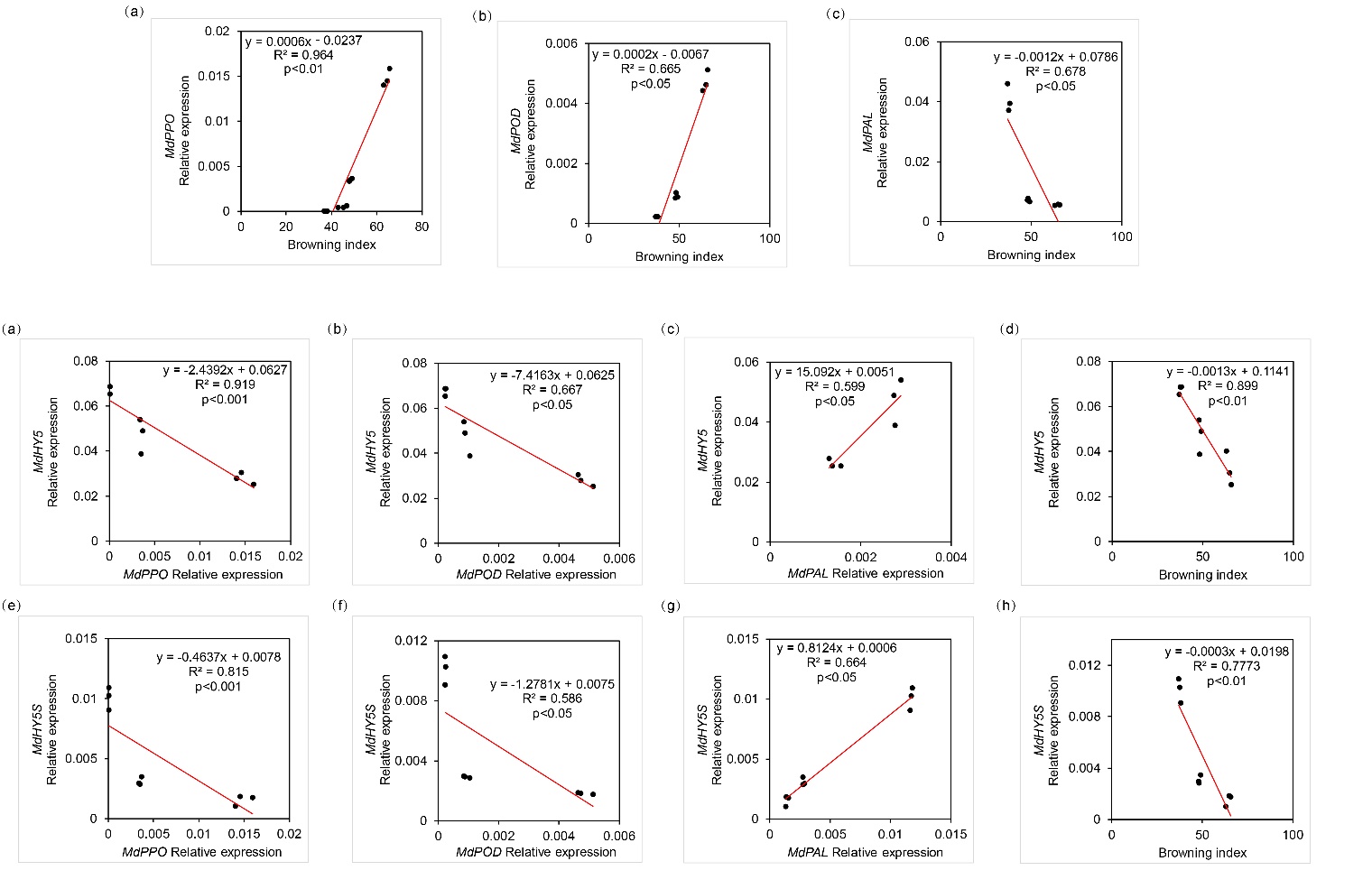


**Supplemental Figure S5.** *MdPPO*, *MdPOD*, and *MdPAL* transcript levels were highly correlated with the browning index.

The correlation between *MdPPO* expression level and browning index under 700 lx violet light (a), the correlation between *MdPOD* expression level and browning index under 700 lx violet light (b), and the correlation between *MdPAL* expression level and browning index under 700 lx violet light (c) were determined by linear regression analysis.

**
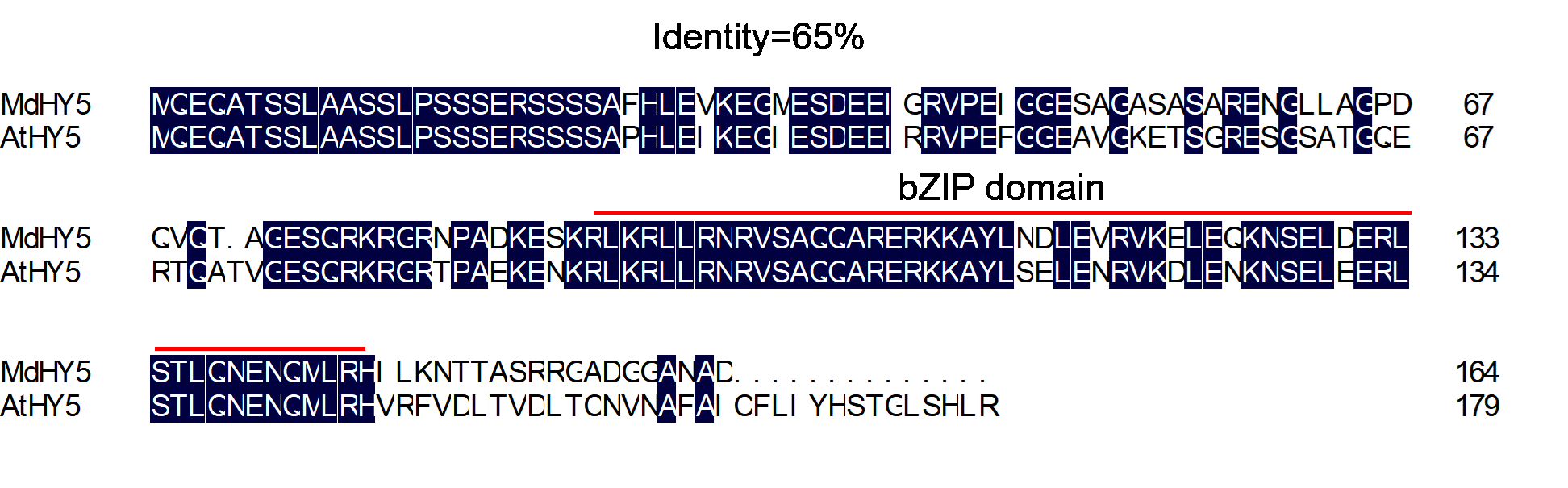
**

**Supplemental Figure S6.** Amino acid sequence alignment of MdHY5 and AtHY5 proteins. ATHY5 represents the HY5 from *Arabidopsis*.

**
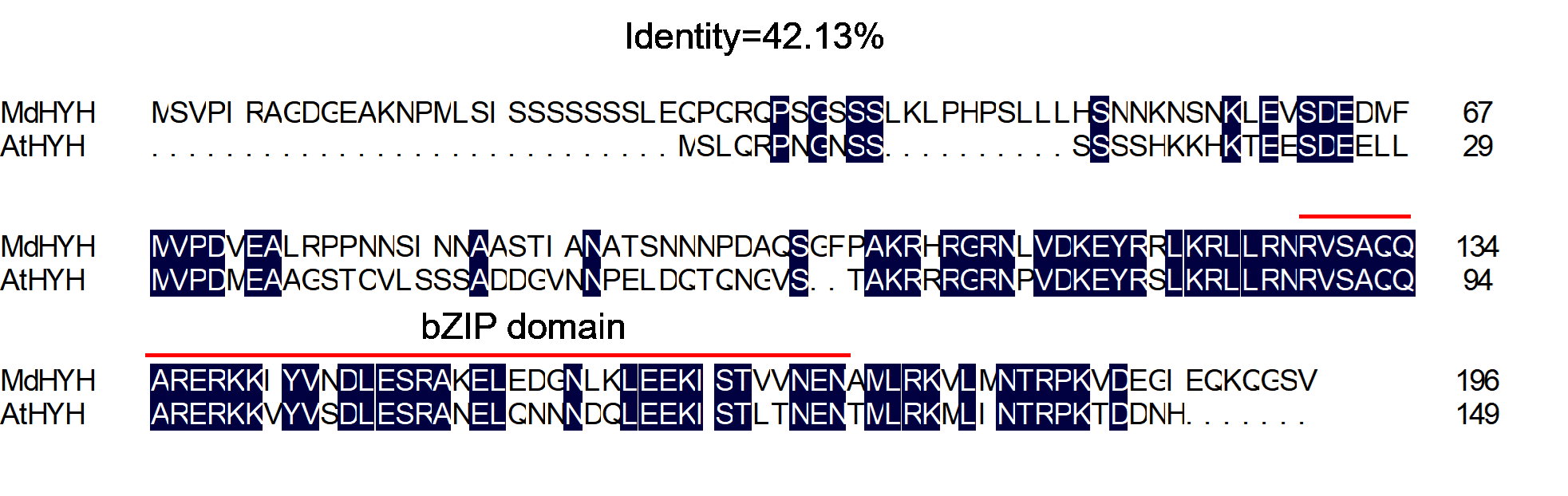
**

**Supplemental Figure S7.** Amino acid sequence alignment of MdHYH and AtHYH proteins. ATHYH represents the HY5 homologous gene from *Arabidopsis*.

**
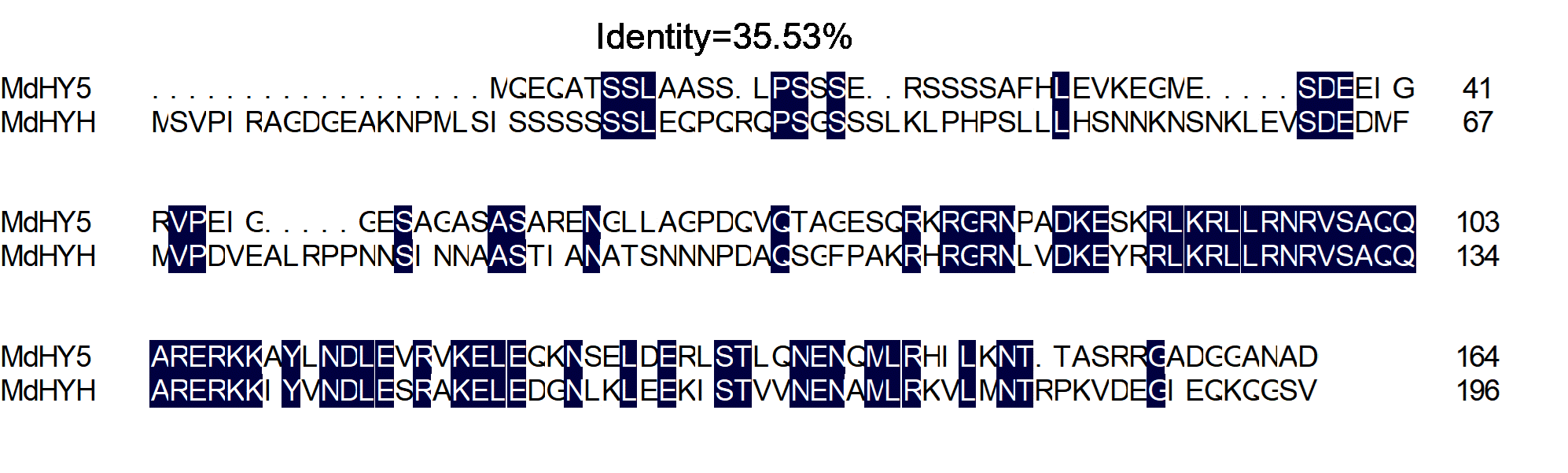
**

**Supplemental Figure S8.** Amino acid sequence alignment of MdHY5 and MdHYH proteins.


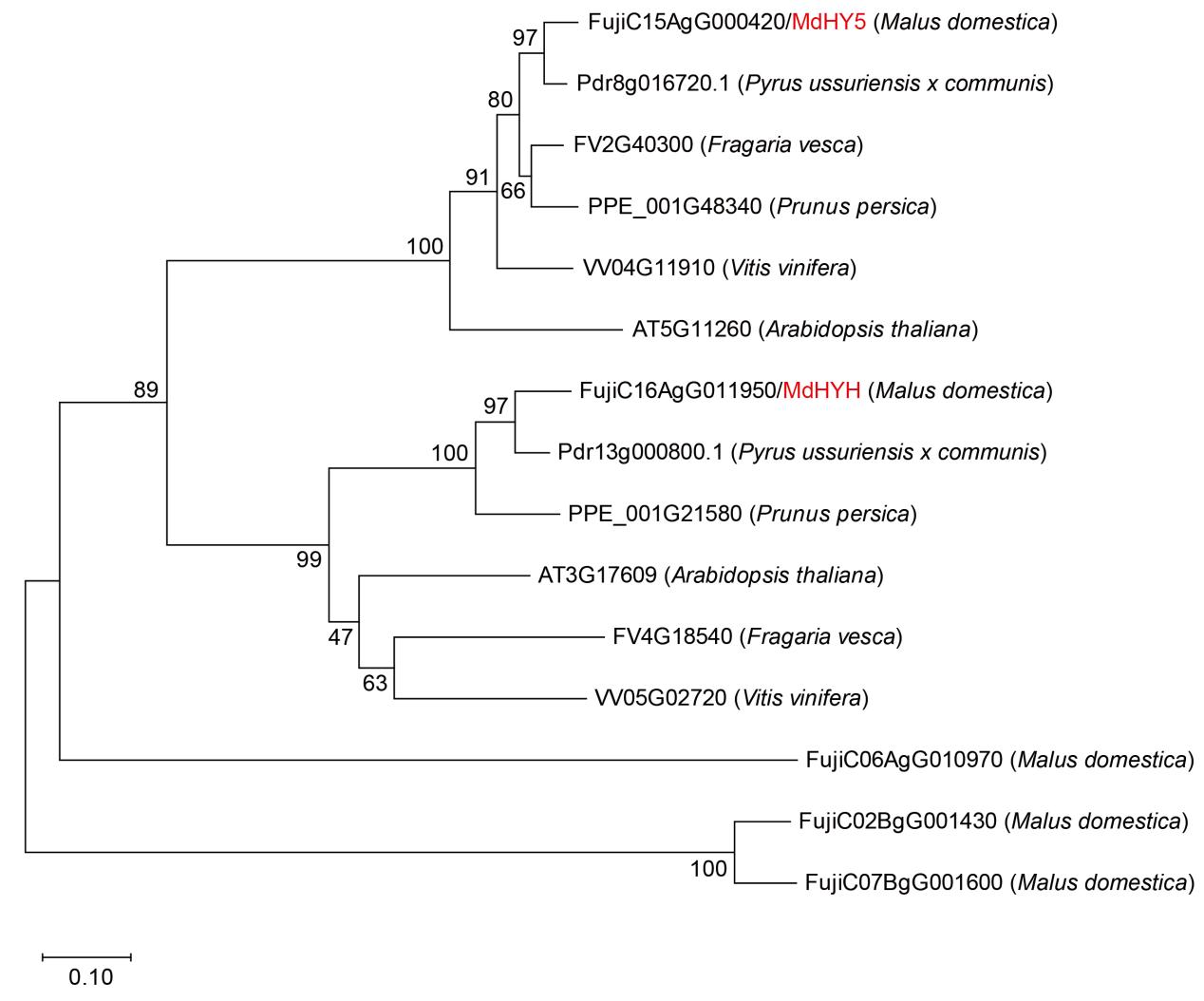


**Supplemental Figure S9.** Phylogenetic tree analysis of MdHYHs and HY5s from different species.

Amino acid sequences of HY5s in different species were aligned using MEGA 11 software. The Bootstrap value was 1000 replicates. Scale bar: 0.1 substitutions per site. The MdHY5 and MdHYH proteins were marked by red.

**
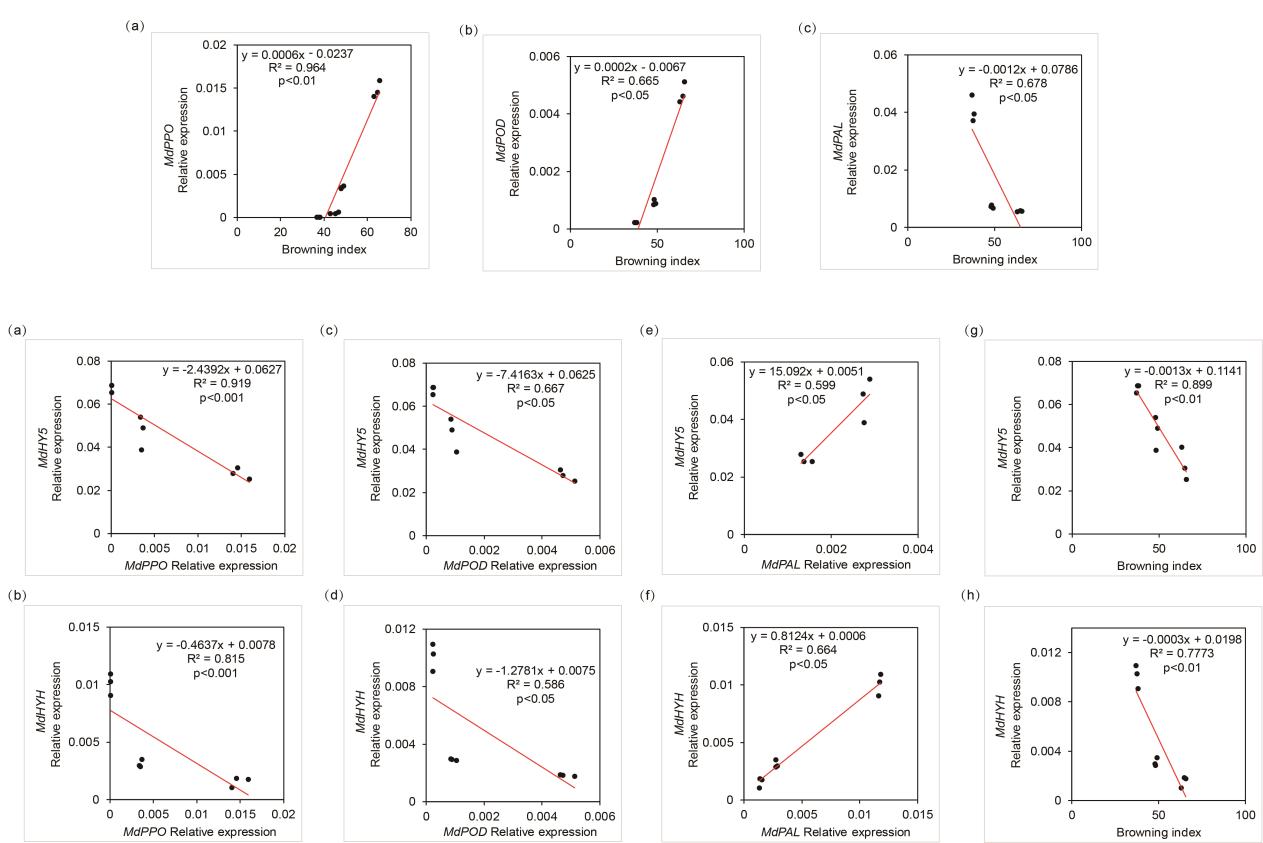
**

**Supplemental Figure S10.** *MdHY5* and *MdHYH* were highly correlated with *MdPPO*, *MdPOD*, *MdPAL* expression levels and browning index.

The correlation between *MdHY5/MdHYH* expression level and *MdPPO* expression level under 700 lx violet light treatment (a, b), the correlation between *MdHY5/MdHYH* expression level and *MdPOD* expression level under 700 lx violet light treatment (c, d), the correlation between *MdHY5/MdHYH* expression level and *MdPPO* expression level under 700 lx violet light treatment (e, f), and the correlation between *MdHY5/MdHYH* expression level and browning index under 700 lx violet light treatment (g, h) were measured by linear regression analysis.


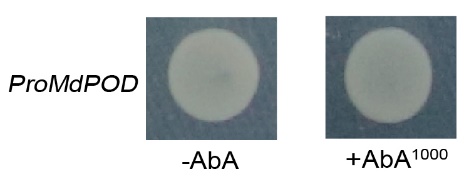


**Supplemental Figure S11.** *ProMdPOD* promoter could not be suppressed by AbA^1000^ on the SD/-Ura medium.

Yeast cells transformed with *ProMdPOD* promoter were grown on the SD/-Ura medium supplemented without AbA (left panel) or with AbA^1000^ (right panel).

**
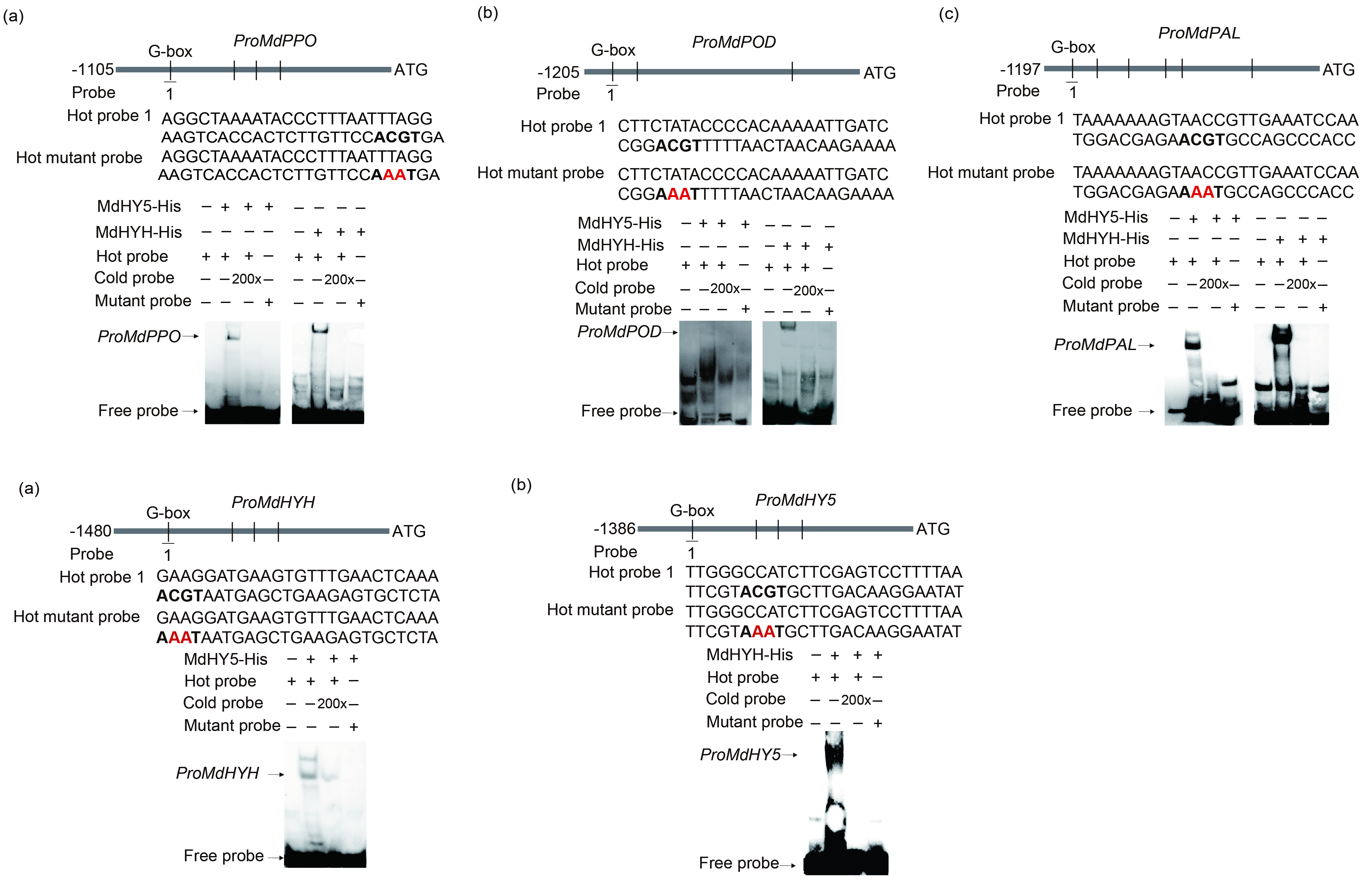
**

**Supplemental Figure S12.** MdHY5 and MdHYH directly bound to *MdPPO, MdPOD,* and *MdPAL* promoters.

The EMSA analysis showed that MdHY5 and MdHYH bound to the *MdPPO* promoter (a), *MdPOD* promoter (b), and *MdPAL* promoter (c) with G-box. The hot probes were designed to represent biotin-labeled *MdPPO, MdPOD,* or *MdPAL* promoters and cold probes were designed to represent unlabeled competitive probes (with a concentration of 200 x). A hot probe with two nucleotide mutations was used as the mutation probe. Mutation sites were marked in red. MdHY5-His and MdHYH-His proteins were purified. The probe sequence is shown above. The ‘‘+’’ or ‘‘-’’ meant presence or absence.

**
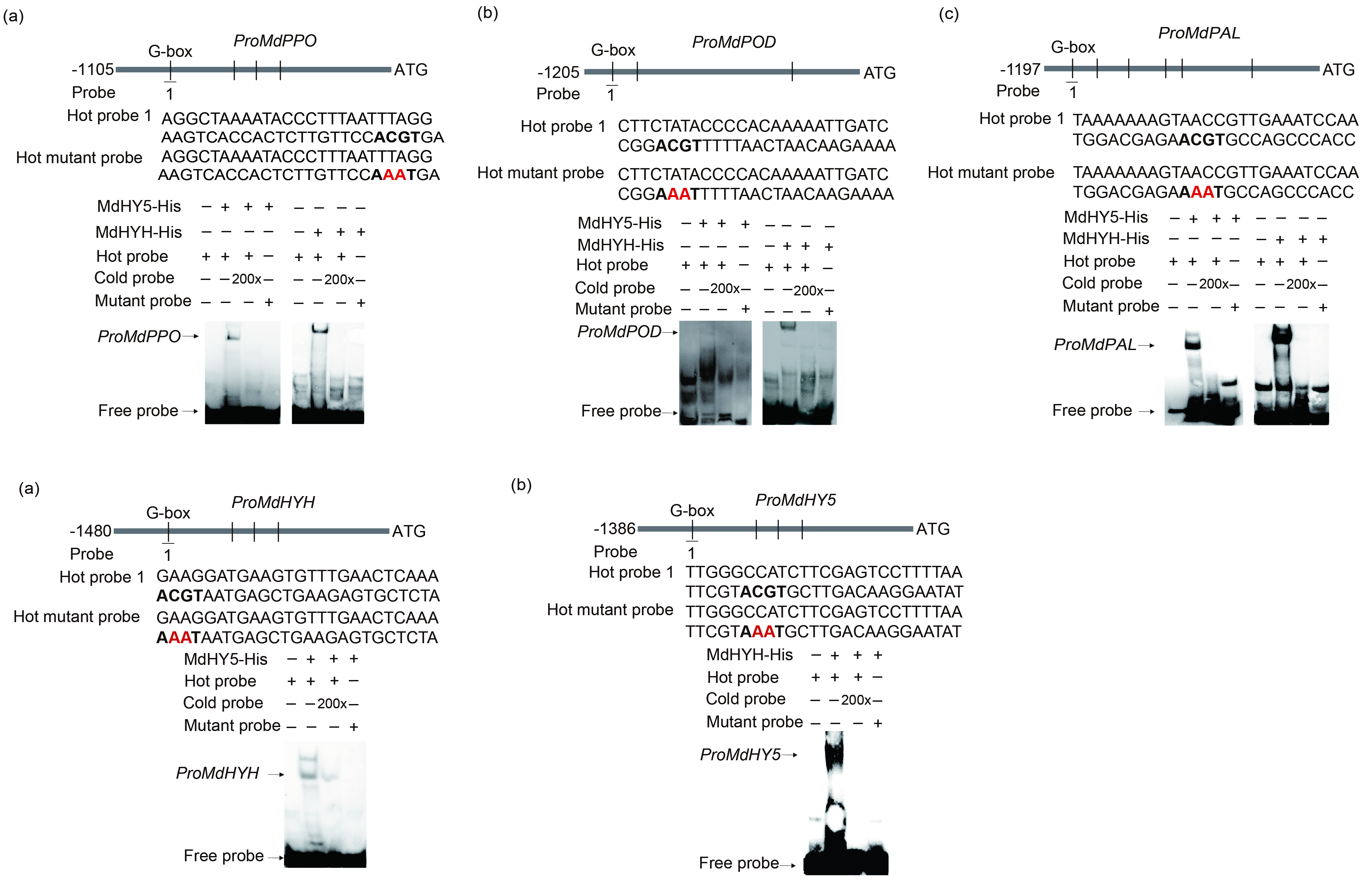
**

**Supplemental Figure S13.** MdHY5 and MdHYH directly bound to their corresponding promoters.

The EMSA analysis showed MdHY5 bound to MdHYH promoter (a), and MdHYH bound to MdHY5 promoter (b) with G-box. The hot probes were designed to represent biotin-labeled *MdHYH* or *MdHY5* promoters, and cold probes were designed to represent unlabeled competitive probes (with a concentration of 200 x). A hot probe with two nucleotide mutations was used as the mutation probe. Mutation sites were marked in red. MdHY5-His and MdHYH-His proteins were purified. The probe sequence is shown above. The ‘‘+’’ or ‘‘-’’ meant presence or absence.
